# Molecular Parasitic Plant–Host Interactions

**DOI:** 10.1371/journal.ppat.1005978

**Published:** 2016-12-15

**Authors:** Simon B. Saucet, Ken Shirasu

**Affiliations:** RIKEN Center for Sustainable Resource Science, Yokohama, Kanagawa, Japan; THE SAINSBURY LABORATORY, UNITED KINGDOM

## Introduction

Throughout evolution, a wide number of organisms specialized in parasitizing plants. Plants are not exceptions; certain plant species evolved as parasites of their own kind. Parasitic angiosperms evolved at least 12 times and show various lifestyles. For example, facultative parasitic plants can complete their life cycle and produce seeds without hosts, whereas obligate parasitic plants totally rely on their hosts. Some obligate parasitic plants, such as broomrapes (*Orobanche* spp.), witchweeds (*Striga* spp.), and dodders (*Cuscuta* spp.), are major crop pathogens that cause severe and persistent damage in agriculture. During parasitism, series of molecular signals are emitted by nearby plants and perceived by parasitic plants. These stimuli are often essential for parasitic plants to germinate and/or undergo parasitic stages in the right place and at the right time. On the other hand, a growing body of evidence supports the idea that plant immunity programs can be activated by detection of molecules derived from parasitic plants. Here, we summarize the molecular interactions between parasitic plants and host plants, mainly obtained from studies on Orobanchaceae parasitic plants.

## How Do Parasitic Plants Sense the Presence of a Potential Host for Germination?

**Fig 1:**
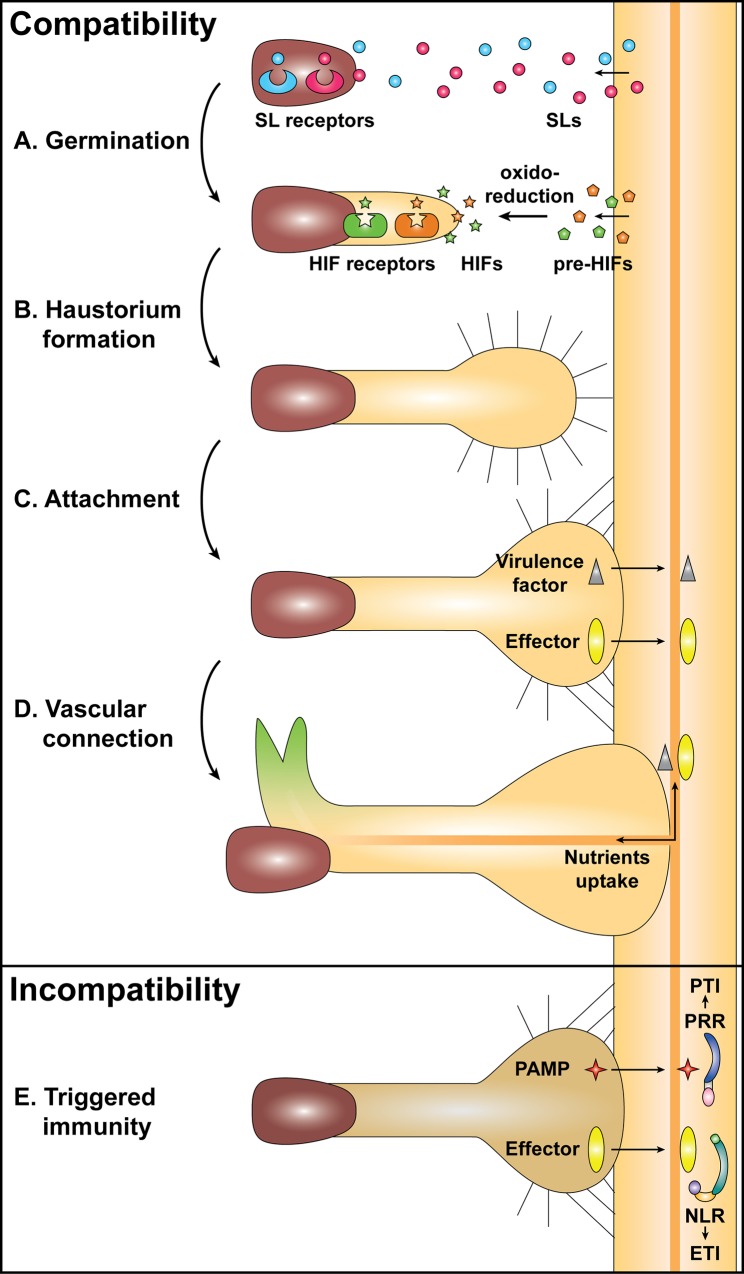
A model for molecular interactions between a parasitic plant and a potential host. An obligate parasitic plant, such as *Striga* spp., is represented on the left side of the figure and a potential host root on the right. The upper box shows the parasitic plant developmental stages (**A**, **B**, **C**, and **D**) associated with the corresponding molecular exchanges that can occur during a compatible interaction. The lower box shows the host-invading stage in which parasitic plant molecules likely trigger immunity in the host (**E**), leading to an incompatible interaction and arrest of parasitism. **A**: The strigolactones (SLs) perception by SL receptors triggers the germination of some parasitic plants, such as *Striga* and *Orobanche* species. **B**: Oxidoreduction processes enable the production of haustorium-inducing factors (HIFs) from precursors of HIFs (pre-HIFs), in some cases. The perception of active HIFs by HIF receptors activates formation of the haustorium, a globular invading organ. **C**: The haustorial hairs and the haustorial growth enable the attachment of the parasitic plant to the host root. **D**: Further growth of the haustorium and probable secretion of unknown virulence factors (i.e., small compounds) and/or effector proteins participate in the instalment of the parasite inside the host root. A vascular connection is finally established with the host root for nutrients uptake. **E**: Parasitic plant-derived molecules, such as pathogen-associated molecular patterns (PAMPs) and effectors, may activate PAMP-triggered immunity (PTI) and effector-triggered immunity (ETI) through their recognition by PAMP recognition receptors (PRRs) and by nucleotide-binding domain and leucine-rich repeat-containing (NLR) receptors, respectively.

Many obligate parasitic Orobanchaceae plants, such as *Striga* spp. and *Orobanche* spp., produce a large quantity of small seeds that can remain dormant in soil for decades. The parasites perceive chemical signals emitted by a nearby host, which can disrupt this dormancy and stimulate germination (**Fig 1A**). On the other hand, facultative Orobanchaceae parasites, such as *Triphysaria versicolor* and *Phtheirospermum japonicum*, do not require host-derived germinating stimulus. As seeds of obligate parasitic plants have limited resources, perception of host-derived germination factors ensures the presence of a potential host at a reachable distance. Most of these germination factors discovered so far are strigolactones (SLs). SLs are carotenoid-derived compounds, which can be released into the root exudates [1]. A large number of SL variants have been identified from various hosts, as well as nonhost plants [2]. SLs have variable germination activities against different Orobanchaceae family members, suggesting an adaptation of the parasites for their hosts [2]. The nature of exuded SLs can be a criterion for crop susceptibility or resistance, due to their impact on germination of the parasites [2, 3].

In many plants, SLs are general phytohormones involved in shoot and roots developmental processes [4, 5] as well as responses to abiotic stresses and biotic interactions [6–8]. SL receptors are α/β-fold hydrolases encoded by *Dwarf 14* (*D14*) orthologs in various plants [9, 10]. In Arabidopsis, there is another related α/β-fold hydrolase homolog encoded by *KARRIKIN INSENSITIVE 2* (*KAI2*) (also known as *D14-LIKE* or *HTL* [for *HYPOSENSITIVE TO LIGHT*]) genes [11]. Interestingly, *KAI2*, but not *D14*, is involved in seed germination in Arabidopsis. Surprisingly, several Orobanchaceae families encode one D14 homolog but multiple KAI2 homologs, suggesting an expansion of the KAI2 receptor family [12–14]. In particular, the SL-binding cavity of KAI2 homologs in Orobanchaceae is structurally different with variable binding affinities to the different SL tested [12]. Thus, it is hypothesized that the expansion of KAI2 homologs in different parasitic plant populations might enable recognition of diverse SLs, leading to an increase and/or specialization of their host range. It would be interesting to know if the Orobanchaceae plants also produce SLs, which SLs they produce, what their physiological roles are, and if the downstream signalling pathways are similar to those in nonparasitic plants.

## How Do Parasitic Plants Initiate Haustorium Formation?

In order to invade the host, parasitic plants need to develop a specialized organ called a haustorium, which attaches to and penetrates into the host root or stem (**Fig 1B**) [15]. Unlike fungi and oomycetes, the haustorium of parasitic plants is a multicellular organ. In some Orobanchaceae plants, root hair-like structures on the surface of the haustorium elongate to capture the host (**Fig 1C**) [16]. Once the host root is penetrated, the haustorium establishes a connection with the host vascular system, enabling the parasite to acquire water and nutrients and to modulate host physiology, probably via secretion of virulence factors such as small molecules and proteins known as effectors (**Fig 1C and 1D**) [17]. In the Orobanchaceae plants, haustorium formation can occur at the meristematic tip of the parasite primary root (called terminal haustorium) or at the transition zone on the side of a growing root (lateral haustorium) [15]. While haustorium formation can be triggered by physical stimulus in some parasitic plants, it often requires the perception of secondary metabolites known as haustorium-inducing factors (HIFs). So far, almost all the natural and synthetic HIFs identified for Orobanchaceae plants are phenolic derivatives, such as flavonoids or quinones [18, 19]. HIF recognition specificities potentially play a role in determining host ranges and in avoiding spontaneous haustorium formation on a parasitic plant’s own roots, as well as nonproductive association (e.g., with congeneric or conspecific plants) [20, 21].

Little is known about the origin of HIFs and how HIFs are perceived by parasitic plants. In the case of Orobanchaceae plants, a speculative model proposed that the production of reactive oxygen species (ROS) by the root parasitic plants convert host-derived phenolic precursors of HIFs, such as syringic acid, into active HIFs, such as 2,6-dimethoxy-*p*-benzoquinone [22, 23]. In this way, parasitic plants may be able to ensure the close proximity of the host for haustorial commitment. Interestingly, by testing structurally different quinones, a correlation was established between their redox potential and their ability to trigger haustorium formation [24]. In addition, combined expression and silencing data showed that a NADPH-dependent oxidoreductase identified in *T*. *versicolor* is involved in quinone-triggered haustorium formation, suggesting that quinones are reduced by the oxidoreductase to produce reactive semiquinones, which activate downstream signalling pathways [25]. However, the mechanism by which semiquinones transduce the signal remains unknown. Hypothetically, this could occur via a ligand–receptor interaction in a structure-dependent manner. The identification of such an HIF receptor represents a crucial step that will shed light on the evolution and adaptation of plant parasitism.

## How Do Parasitic Plants Trigger Immune Responses in the Host?

While parasitic plants hijack host-derived signals to initiate and synchronize their parasitic stages, resistant plants have developed defence mechanisms activated upon the parasite attack (**Fig 1E**). Various defence responses have been observed, such as induction of immunity-related genes, ROS production, deposition of callose and other phenolic compounds, and vessel occlusion, as well as localized hypersensitive response (HR), which often leads to the arrest of the invasion followed by the necrosis of the parasitic structures [26–28]. The nature and timing of the defence responses together with the molecular components involved suggest the implication of the multilayered plant innate immune system originally described to function against microorganisms [29]. This surveillance system deploys receptors that activate defence signalling upon perception of molecular determinants from pathogens. In the context of plant–microbe interactions, cell surface pattern recognition receptors (PRR) bind to pathogen-associated molecular patterns (PAMPs), while nuclear and/or cytoplasmic resistance (R) receptors sense pathogen-secreted effector proteins.

The involvement of immune receptors became a tempting hypothesis when a diffusible proteinaceous signal, as a potential PAMP, from germinated *Orobanche ramosa* was shown to trigger defence responses in Arabidopsis cells [30]. Recently, a PRR called CuRe1 that recognizes a potential PAMP from the stem of the parasitic plant *Cuscuta reflexa* was identified in tomatoes, suggesting that a PAMP from parasitic plants can be recognized by other plants, supporting such a hypothesis [31]. In addition, the discovery of cowpea *R* gene *RSG3-301* against *Striga gesnerioides*, encoding a nucleotide-binding domain and leucine-rich repeat-containing (NLR) receptors, brought the first piece of evidence that an immune receptor can recognize a parasitic plant [32]. In this case, the recognition occurred in the cortex and triggered an HR, which is often observed during pathogen effector-triggered NLR–mediated immunity. This may suggest that parasitic plants may secrete effector proteins during infection, but more importantly, it highlights future prospects in the identification of more NB-LRR *R* genes against parasitic plants. Nevertheless, it remains essential to determine the PAMP and the effector recognized by CuRe1 and RSG3-301, respectively. The identification and characterization of parasitic plant-derived molecules and their cognate receptors will provide a significant step towards the prediction and elaboration of resistance in crops.

## Concluding Remarks

Due to the large diversity of parasitic plants and their equally diverse infection strategies, the molecular interactions occurring with their different hosts must vary. However, it is clear that the outcome of parasitic plant–host interactions is directly linked with the ability of each protagonist to perceive the other as a nonself; parasitic plants sense potential host molecules for germination and haustorium formation, while resistant plants detect parasite molecules for immune system activation. A next challenge is to address to what extent parasitic plant-derived molecules perform virulence function, such as host immunity suppression, for example. In addition, further identification of immune components against parasitic plants might reveal specific defence mechanisms against this type of pest. As ligand–receptor interactions must apply high pressure on the parasite–host co-evolution, future studies on this subject should unveil the dynamic nature of adaptation, as well as speciation, resulting from parasitic plant–host interactions.
